# The Risk of Colorectal Adenoma in Nonalcoholic or Metabolic-Associated Fatty Liver Disease

**DOI:** 10.3390/biomedicines9101401

**Published:** 2021-10-05

**Authors:** Ji-Yeon Seo, Jung-Ho Bae, Min-Sun Kwak, Jong-In Yang, Su-Jin Chung, Jeong-Yoon Yim, Seon-Hee Lim, Goh-Eun Chung

**Affiliations:** Department of Internal Medicine and Healthcare Research Institute, Seoul National University Hospital Healthcare System Gangnam Center, 39FL., Gangnam Finance Center 737, Yeoksam-dong, Gangnam-gu, Seoul 135-984, Korea; sjy@snuh.org (J.-Y.S.); bjh@snuh.org (J.-H.B.); kms39@snuh.org (M.-S.K.); drmirinae@snuh.org (J.-I.Y.); medjsj@snuh.org (S.-J.C.); yjy@snuh.org (J.-Y.Y.); limsh@snuh.org (S.-H.L.)

**Keywords:** hepatic steatosis, adenoma, metabolic dysfunction, fibrosis

## Abstract

Nonalcoholic fatty liver disease (NAFLD) is the most common cause of liver disease associated with various metabolic disorders. Metabolic dysfunction-associated fatty liver disease (MAFLD) emphasizes metabolic dysfunction in NAFLD. Although the relationship between NAFLD and colorectal adenomas has been suggested, the effect of MAFLD on colorectal adenoma has yet to be investigated. In this study, we examined the relationship between NAFLD/MAFLD and colorectal adenoma in comparison with other metabolic factors. Methods: Examinees who underwent colonoscopy and abdominal ultrasonography on the same day from January 2012 to December 2012 were included. NAFLD was diagnosed according to the findings of ultrasonography. The Fibrosis-4 (FIB-4) index was used as a surrogate marker for advanced hepatic fibrosis. A logistic regression model was used to analyze the risk of NAFLD/MAFLD for colorectal adenoma. Results: The prevalence of NAFLD and MAFLD was 37.5% and 32.8%, respectively. In the multivariate analysis, male sex, older age, diabetes, and smoking increased the risk of colorectal adenoma. NAFLD and MAFLD were the most important risk factors for colorectal adenoma only in females [adjusted odds ratio (OR) 1.43 and 95% confidence interval (CI) 1.01–2.03, and OR 1.55, 95% CI 1.09–2.20, respectively]. NAFLD and MAFLD with an advanced fibrosis index were significantly associated with an increased risk of colorectal adenoma. (NAFLD: OR 1.38, 95% CI, 1.04–1.83, *p* = 0.027; MAFLD: OR 1.45, 95% CI, 1.13–1.96, *p* = 0.004, respectively). Conclusion: NAFLD and MAFLD were significantly associated with a higher risk of colorectal adenomas, especially in females. NAFLD and MAFLD with advanced fibrosis were associated with an increased risk of colorectal adenoma. Colonoscopic examinations may be emphasized for patients with NAFLD/MAFLD, for women, or patients with the presence of hepatic fibrosis.

## 1. Introduction

Nonalcoholic fatty liver disease (NAFLD) is the most common cause of chronic liver disease worldwide, with an increasing prevalence of up to 20–30% [1]. Although NAFLD is generally a benign condition, some patients may progress to nonalcoholic steatohepatitis (NASH), fibrosis, and cirrhosis [2]. Hepatic steatosis is commonly associated with various metabolic conditions, including cardiovascular disease [3], diabetes [4], chronic kidney disease [5], and colorectal cancer (CRC) [6]. Recently, the new definition of ‘metabolic (dysfunction)-associated fatty liver disease (MAFLD)’ has been introduced, with an emphasis on the role of metabolic dysfunction in the clinical outcomes of patients with fatty liver disease [7,8].

CRC is the third most commonly diagnosed cancer and the fourth most common cause of cancer-related deaths worldwide [9]. Colorectal adenoma has been well known as a precursor lesion of CRC, and accumulation of mutation and metabolic reprogramming starts at the adenoma stage [10]. Since NAFLD and colorectal adenoma share proinflammatory conditions and metabolic risk factors, such as diabetes, obesity, and insulin resistance [11,12,13,14], the association between NAFLD and colorectal neoplasms has been investigated [15,16]. A systemic review reported that NAFLD was associated with a moderately increased prevalence and incidence of colorectal adenomas [17]. Until now, studies on the risk of colorectal neoplasia in terms of both NAFLD and MAFLD have been limited. A recent study reported that nonobese MAFLD, rather than NAFLD, was the most important factor associated with the presence of colorectal adenoma [18]. However, studies included a relatively small number of subjects, and whether MAFLD is a possible new risk factor for colorectal adenoma remains uncertain.

Thus, we aimed to investigate the relationship of colorectal adenoma and both NAFLD and MAFLD in asymptomatic individuals undergoing screening colonoscopy. We also evaluated whether the severity of NAFLD/MAFLD was associated with the risk of these neoplasms.

## 2. Methods

### 2.1. Study Population

We used a previously conducted retrospective cohort [19]. Briefly, from January 2012 to December 2012, subjects who underwent routine health checkup at the Seoul National University Hospital Healthcare System Gangnam Center were consecutively enrolled. Among them, we included subjects who underwent abdominal ultrasonography and screening colonoscopy on the same day, and a total of 3441 subjects were included in the analysis.

For NAFLD analysis, subjects who displayed any potential cause of chronic liver disease were excluded; 149 were positive for the hepatitis B virus, 29 were positive for the hepatitis C virus, and 219 had significant alcohol intake (>20 g/day for males and >10 g/day for females) [20]. As a result, 3044 subjects were included in the NAFLD analysis.

The study protocol was approved by the Institutional Review Board of Seoul National University Hospital (IRB No. H-2106-014-1223) and conformed to the ethical guidelines of the World Medical Association Declaration of Helsinki. The requirement for informed consent from individual subjects was waived, as we used de-identified secondary data.

### 2.2. Clinical and Biochemical Evaluations

Study data consisted of medical information based on a self-administered questionnaire and anthropometric and laboratory measurements as previously described [19]. In brief, waist circumference (WC) was measured at the midpoint between the lower costal margin and the anterior superior iliac crest by a well-trained person using a tape measure. Height and body weight were measured using a digital scale. Body mass index (BMI) was calculated as weight (kg)/height^2^ (m^2^). Obesity was defined as a BMI ≥ 25 (kg/m^2^), according to the World Health Organization recommendation for the Asia–Pacific region [21]. Based on smoking status, subjects were categorized as current smokers or noncurrent smokers. Blood pressure was measured at least twice, and the mean values of the measurements were recorded. Hypertension was defined as a blood pressure ≥140/90 mmHg or a history of receiving antihypertensive medications. Diabetes was defined as fasting glucose levels ≥ 126 mg/dL, a glycated hemoglobin level (HbA1c) ≥ 6.5%, or a history of receiving glucose-lowering agents.

All subjects fasted for at least 12 h prior to blood sampling; aspartate aminotransferase (AST), alanine aminotransferase (ALT) total cholesterol, triglyceride, high-density lipoprotein (HDL) cholesterol, glucose, HbA1c, and high-sensitivity C-reactive protein (CRP) levels were measured. All tests were performed using standard laboratory methods. We used a previously described method to assess the visceral fat area (VFA) on abdominal fat computed tomography (CT) images [22]. The individuals were examined in the supine position with a 16-detecter CT scanner (Somatom Sensation 16; Siemens Medical Solutions, Forchheim, Germany). The VFA was measured using commercially available CT software (Rapidia 2.8; INFINITT, Seoul, Korea) by setting the attenuation values for adipose tissue areas with a range of −250 to −50 Hounsfield units.

### 2.3. Diagnosis of NAFLD/MAFLD and Advanced Fibrosis

Hepatic ultrasonography (Acuson Sequoia 512; Siemens, Mountain View, CA, USA) was performed to diagnose fatty liver disease by experienced radiologists who were unaware of the clinical information of the subjects [23]. Fatty liver disease was diagnosed based on characteristic ultrasonographic findings consistent with a ‘‘bright liver’’ and evident contrast between hepatic and renal parenchyma, focal sparing, vessel blurring, and narrowing of the lumen of the hepatic veins [24]. The diagnosis of MAFLD was based on previous criteria [8]. For subjects with NAFLD or MAFLD, the Fibrosis-4 (FIB-4) index was used as a surrogate marker for advanced liver fibrosis, and an FIB-4 index score of less than 1.3 excluded advanced liver fibrosis with high accuracy [25].

### 2.4. Colonoscopic Examination

All colonoscopic examinations were performed by using a video colonoscopy system (FICE 4400 series; Fujinon, Saitama, Japan or CF-H260 series; Olympus Optical Co, Tokyo, Japan) by board-certified gastroenterologists who had performed more than 5000 colonoscopies [26]. All endoscopists were blinded to the laboratory findings at the time of the procedure. The surveillance intervals were determined based on the baseline endoscopic and histopathological findings according to the guidelines suggested by the U.S. Multi-Society Task Force on Colorectal Cancer and the Korean guidelines for colorectal cancer screening and postpolypectomy colonoscopy surveillance [27,28]. For adequate bowel preparation, subjects were given 4 L of polyethylene glycol lavage solution (Colonlyte®; Meditech Korea Pharmacy, Seoul, Korea). During the colonoscopy, either intravenous midazolam and pethidine or pethidine alone was administered by the gastroenterologist according to the medical condition of the participant. The colonoscope reaching the cecum, documented by a picture of the ileocecal valve, was defined as a complete colonoscopic examination, which was higher than 98%. All polypoid lesions were biopsied or removed and histologically assessed by experienced pathologists according to the WHO criteria [29].

### 2.5. Statistical Analyses

Data are presented as the mean ± standard deviation for normally distributed continuous variables and as proportions for categorical variables. Student’s t-test and analysis of variance were used to analyze continuous variables, and the differences between nominal variables were compared with the chi-square test. A logistic regression analysis was utilized to analyze the association between NAFLD or MAFLD and colorectal adenomas after adjusting for potential confounders. Among variables with a *p* value of less than 0.05 in the univariate analysis, those with clinical importance were subjected to multivariate analyses. All statistical analyses were performed using SPSS 22.0 (SPSS Inc., Chicago, IL, USA), and *p* values < 0.05 were considered statistically significant.

## 3. Results

### 3.1. Baseline Characteristics of the Study Population

Among the 3441 subjects, the mean age was 52.4 years, and the male proportion was 61.9%. The prevalence rate of colorectal adenoma was 29.4%. The baseline characteristics of the study participants according to NAFLD or MAFLD are shown in Table 1. NAFLD or MAFLD were more frequently observed in people who were male and current smokers. The prevalence of diabetes and hypertension was significantly higher in subjects with NAFLD or MAFLD. Additionally, most of the anthropometric and laboratory variables (including BMI, WC, systolic or diastolic blood pressure, and AST, ALT, total cholesterol, triglyceride, and HDL cholesterol levels) were less metabolically favorable in subjects with NAFLD or MAFLD (*p* < 0.001). The prevalence of colorectal adenoma was significantly higher in both NAFLD and MAFLD patients than in those without NAFLD/MAFLD. Baseline characteristics stratified by sex are provided in Supplementary Materials Tables S1 and S2.

### 3.2. Risk of Colorectal Adenoma in Subjects with NAFLD/MAFLD

We investigated the association between colorectal adenomas and NAFLD/MAFLD. In the univariate model, the presence of NAFLD showed a significant association with colorectal adenoma. NAFLD was associated with a 45% increased risk of colorectal adenomas (odds ratio [OR] 1.45, 95% confidence interval [CI] 1.24–1.70, *p* < 0.001). After adjusting for age, sex, BMI, diabetes, hypertension, smoking, triglyceride level, HDL cholesterol level, and VFA, the statistical significance of NAFLD disappeared (OR 1.12, 95% CI, 0.92–1.35, *p* = 0.256). Moreover, diabetes and smoking showed significant associations with an increased risk of colorectal adenoma (OR 1.45, 95% CI, 1.12–1.91, *p* = 0.005 and OR 1.35, 95% CI, 1.05–1.74, *p* = 0.020, respectively, Table 2). MAFLD was also statistically associated with a significant risk of colorectal adenoma in the univariate analysis (OR 1.31, 95% CI 1.12–1.53, *p* = 0.001); however, there was no significant association between MAFLD and colorectal adenoma in the multivariate analysis (OR 1.08, 95% CI 0.91–1.28, *p* = 0.409).

When we performed stratified analysis according to sex, NAFLD was independently associated with the risk of colorectal adenoma in women (OR 1.43, 95% CI, 1.01–2.03, *p* = 0.046), while diabetes and smoking showed significant associations with an increased risk of colorectal adenoma in men in the multivariate analysis (OR 1.50, 95% CI, 1.11–2.03, *p* = 0.008 and OR 1.40, 95% CI, 1.07–1.83, *p* = 0.014, respectively). MAFLD was independently associated with the risk of colorectal adenoma in women (OR 1.55, 95% CI, 1.09–2.20, *p* = 0.015, Table 3). In contrast, smoking showed an increased risk of colorectal adenoma in men with MAFLD (OR 1.38, 95% CI 1.09–1.74, *p* = 0.007).

### 3.3. The Relationship of Colorectal Adenoma and NAFLD with Diabetes or Obesity

Since diabetes and obesity are both well-known confounders and significant risk factors for colorectal adenoma, we evaluated the relationship between colorectal adenoma and NAFLD with diabetes or obesity. After adjusting for age, sex, smoking, triglyceride level, HDL cholesterol level, hypertension, VFA, and BMI, the NAFLD-with-diabetes group had a significantly increased risk for colorectal adenoma compared with the no-NAFLD group (OR 1.75, 95% CI, 1.25–2.45, *p* = 0.001). However, there was no statistical significance in the increased risk of the NAFLD-with-obesity group compared with the no-NAFLD group. When we compared the MAFLD group with the no-fatty liver disease group, there was no significant difference in the risk for colorectal adenoma. Moreover, when we further divided the MAFLD group into three categories according to BMI and diabetes, the proportion of colorectal adenoma was the highest in the MAFLD-with-diabetes group; however, there was no significant difference regarding the risk of colorectal adenoma in the multivariate analysis, as displayed in Table 4.

### 3.4. Subgroup Analysis in Subjects with NAFLD/MAFLD

Next, we evaluated the association of advanced fibrosis and colorectal adenoma in individuals with NAFLD or MAFLD. When participants with NAFLD/MAFLD were grouped according to advanced fibrosis using the FIB-4 index, those who had advanced fibrosis with high FIB-4 index scores showed an independently increased risk for colorectal adenoma compared with those who had low FIB-4 index scores (NAFLD; OR 1.38, 95% CI, 1.04–1.83, *p* = 0.027 and MAFLD; OR 1.45, 95% CI, 1.13–1.96, *p* = 0.004, respectively, Table 5).

## 4. Discussion

In the present study, the presence of NAFLD and MAFLD was significantly associated with colorectal adenoma in women compared with the subjects in the nonfatty liver disease group, while diabetes and smoking were significantly associated with colorectal adenoma in men after adjusting for confounding factors. In addition, advanced fibrosis was significantly associated with colorectal adenoma in both NAFLD and MAFLD patients.

Colorectal adenoma detection rates (ADRs) during colonoscopy vary widely among endoscopists and are therefore an important factor to consider when comparing adenoma prevalence between groups [30]. The overall ADR in our study was 29%, in line with the expected adenoma detection rate of 20–30% on screening and surveillance colonoscopies [31]. A previous study performed in Korea showed that ultrasound-diagnosed NAFLD was associated with an increased risk of colorectal adenomatous polyps, showing an OR of 1.28 [15]. The risk for colorectal adenomatous polyps was the highest in subjects with both NAFLD and metabolic syndrome in a previous study. Ahn et al. reported that the presence and severity of NAFLD were closely associated with any colorectal neoplasia and advanced colorectal neoplasia in the Korean population [16]. Recently, Blackett et al. found that patients with biopsy-proven NAFLD had a significantly higher adenoma prevalence than matched controls (40.7 vs. 28.1%) [32]. However, Touzin et al. reported that there was no association between biopsy-proven NAFLD and the prevalence of colorectal adenomas [33].

In this study, there was no difference in the risk of colorectal adenomas in those with either NAFLD or MAFLD among the total subjects. In contrast to our results, a recent study performed in Japan reported that nonobese MAFLD was the most important factor associated with the presence of colorectal adenomas, with an increased OR of 3.19 [18]. The strength of the previous study is its histological evaluation of NAFLD; however, one limitation is its relatively small study population. Although the study subjects were enrolled among the health check-up examinees, the median prevalence of diabetes, hypertension, and colorectal adenoma was higher than those of our study (17.7, 25.0, and 37.9%, vs. 9.6%, 21.8%, and 29.4%, respectively), reflecting the differences in the baseline characteristics of the study population that may have caused the different results.

NAFLD is a sexually dimorphic disease with regard to epidemiological and clinical features, including liver-related outcomes [34]. To analyze whether sex influences the association of NAFLD/MAFLD with colorectal adenoma, we presented data classified according to sex. As a result, NAFLD/MAFLD showed a significant association with increased colorectal adenoma only in women, while diabetes and smoking showed a significant association with colorectal adenoma, suggesting that sexual dimorphism extends to extrahepatic manifestations and complications of NAFLD. In contrast to our results, a previous meta-analysis reported that NAFLD was significantly associated with a higher risk of colorectal polyps in males (OR = 1.47; 95% CI = 1.29–1.67) but not in females (OR = 0.88; 95% CI = 0.60–1.29) [35]. Although the reason for different results among studies remains uncertain, a possible explanation is that the higher prevalence of comorbidities, such as hypertension (73.1% vs. 26.9%) and diabetes (77.0% vs. 23.0%), in men compared to women may have affected the risk of colorectal adenoma in addition to NAFLD (65.0% vs. 24.0%). Additionally, residual confounding by some unmeasured factors also contributed to the differences among the studies.

The sex-specific association of colorectal adenoma and NAFLD/MAFLD in our study may possibly be related to sexual dimorphism in NAFLD. Women who are overweight or obese before menopause have a lower risk of hepatic steatosis and have a higher hepatic insulin sensitivity than men, which might contribute to a more beneficial cardiometabolic effect in women. Since this effect seems to be related to estrogen concentrations and alterations in adipose tissue distribution, these processes might change with menopause, progressing adiposity, and worsening hepatic insulin resistance [36]. In a recent systematic review, women had a lower risk of NAFLD than men but a higher risk of progression than men, especially after age 50 [37]. During the menopausal transition, decreases in estrogen lead to a shift in adipose tissue deposition to a visceral distribution, resulting in metabolic sequelae, such as increases in diabetes and atherogenic dyslipidemia among postmenopausal women [38]. Although menopausal status could not be evaluated in this study, the women were significantly older than the men (mean age 52.9 vs. 52.1, *p* < 0.05) in this study, which supports this explanation.

Waist circumference, obesity, lipid profiles, glucose, and hypertension are known factors that play roles in the process of colorectal adenoma [39]. Among these factors, we further performed stratified analysis according to obesity and diabetes. While NAFLD showed no significant association with colorectal adenoma, the NAFLD-with-diabetes group had a significantly increased risk for colorectal adenoma compared with the no-NAFLD group. Consistent with our results, diabetes was proven to be an independent risk factor for colorectal adenoma [40]. In this study, individuals with advanced fibrosis defined by the FIB-4 index were significantly associated with increased colorectal adenoma in both NAFLD and MAFLD patients, in agreement with previous studies [32,41], and this approach is in line with the implementation principles of precision medicine in NAFLD/MAFLD [42].

To our knowledge, this is the largest study to have examined the relationship between MAFLD and colorectal adenomas. In addition, we evaluated multiple metabolic confounding factors, including VFA, by utilizing CT. However, this study has several limitations. First, due to its observational study design, cautious interpretation is required. Second, although liver biopsy is considered to be the gold standard for the diagnosis of NAFLD, it was assessed only by ultrasonography in this study. Ultrasonography has limitations, including interpersonal variability, inaccuracy in patients with low liver fat contents, and the inability to quantify fibrosis [43]. However, liver biopsy is not typically used in asymptomatic individuals in clinical practice, and radiographic techniques, such as ultrasonography or magnetic resonance imaging, are used for the diagnosis of NAFLD. Third, although MAFLD and NAFLD are not entirely equivalent conditions [44], and the FIB-4 index was initially described to predict fibrosis in patients with NAFLD [25], we used the FIB-4 index in both patients with MAFLD and NAFLD. Fourth, although ADR might be influenced by interval and previous colonoscopy results, this study included subjects who underwent screening or surveillance colonoscopy. However, the ADR was comparable with that of other studies involving only first-time examinees. Last, since this study comprised health check-up participants who are likely to be highly motivated to improve their health for any reason, they might not be representative of the general population and thus might result in selection bias. Indeed, the proportion of advanced adenomas was relatively low at 2.4% in this study population. In addition, South Koreans show disparities in the prevalence, location, and shape characteristics of colorectal neoplasia compared to Westerners, and the generalization of our results to other ethnicities might be limited.

In conclusion, we demonstrated that NAFLD and MAFLD were significantly associated with colorectal adenoma in women. NAFLD and MAFLD with advanced fibrosis showed an increased risk of colorectal adenoma. Therefore, clinicians should be aware of the increased risk of colorectal adenoma in NAFLD patients, especially for women. Further experimental observations that could be conducted to define genetic and sex differences in populations with different eating habits are needed.

## Figures and Tables

**Table 1:** Comparison of subjects’ baseline characteristics according to NAFLD and MAFLD.

	No NAFLD (N = 1901)	NAFLD (N = 1143)	*p* Value	No MAFLD (N = 2314)	MAFLD (N = 1127)	*p* Value
Age (years)	52.5 ± 9.3	53.2 ± 8.7	0.041	52.3 ± 9.3	52.8 ± 8.7	0.083
Male, n (%)	946 (49.8)	869 (76.0)	<0.001	1233 (53.3)	897 (79.6)	<0.001
Smoker, n (%)	193 (10.2)	174 (15.2)	<0.001	279 (12.1)	196 (17.4)	<0.001
Hypertension, n (%)	313 (16.5)	329 (28.8)	<0.001	397 (17.2)	354 (31.4)	<0.001
Diabetes, n (%)	110 (5.8)	186 (16.3)	<0.001	123 (5.3)	209 (18.5)	<0.001
BMI (kg/m^2^)	22.5 ± 2.7	25.1 ± 2.7	<0.001	22.6 ± 2.7	25.6 ± 2.5	<0.001
BMI ≥ 25 (kg/m^2^), n (%)	338 (17.8)	568 (49.7)	<0.001	411 (17.8)	632 (56.1)	<0.001
WC (cm)	81.8 ± 7.8	89.4 ± 7.3	<0.001	82.1 ± 7.8	90.7 ± 6.7	<0.001
SBP (mmHg)	114.8 ± 13.7	119.6 ± 12.9	<0.001	115.0 ± 13.7	120.8 ± 12.7	<0.001
DBP (mmHg)	72.8 ± 10.3	77.0 ± 9.7	<0.001	73.2 ± 10.4	78.1 ± 9.8	<0.001
AST (IU/L)	25.5 ± 14.8	29.2 ± 17.5	<0.001	26.0 ± 15.7	29.9 ± 17.6	<0.001
ALT (IU/L)	23.9 ± 17.8	35.3 ± 24.5	<0.001	24.8 ± 18.0	36.5 ± 24.9	<0.001
Total cholesterol (mg/dL)	194.4 ± 34.0	201.2 ± 39.4	<0.001	194.9 ± 34.0	200.8 ± 39.5	<0.001
Triglyceride (mg/dL) +	71 (50,100)	118 (81,161)	<0.001	71 (50,102)	120 (82,165)	<0.001
HDL-cholesterol (mg/dL)	53.7 ± 11.4	47.3 ± 9.2	<0.001	53.5 ± 11.2	46.7 ± 9.0	<0.001
Fasting glucose (mg/dL)	91.9 ± 16.7	102.4 ± 23.7	<0.001	92.0 ± 16.1	104.0 ± 24.5	<0.001
HbA1c (mg/dL)	5.6 ± 0.5	5.9 ± 0.8	<0.001	5.6 ± 0.5	6.0 ± 0.9	<0.001
CRP (mg/dL)	0.2 ± 0.8	0.2 ± 0.9	0.097	0.2 ± 0.8	0.2 ± 0.9	0.135
Visceral fat area (cm^2^)	80.8 ± 24.7	104.3 ± 22.5	<0.001	81.7 ± 24.7	103.8 ± 22.7	<0.001
Adenoma, n (%)	500 (26.3)	390 (34.1)	<0.001	636 (27.5)	374 (33.2)	0.001

Data are shown as the mean ± SD. ⁺ median (interquartile range). NAFLD, nonalcoholic fatty liver disease; MAFLD, metabolic dysfunction-associated fatty liver disease; BMI, body mass index; WC, waist circumference; SBP, systolic blood pressure; DBP, diastolic blood pressure; AST, aspartate aminotransferase; ALT, alanine aminotransferase; HDL, high-density lipid cholesterol; HbA1C, glycated hemoglobin; CRP, c-reactive protein.

**Table 2:** Univariate and multivariate analyses of the risk for colorectal adenoma.

	Univariate OR (95% CI)	*p* Value	Multivariate OR (95% CI)	*p* Value
**NAFLD**				
Age	1.05 (1.04–1.06)	<0.001	1.05 (1.04–1.06)	<0.001
Sex	1.84 (1.56–2.18)	<0.001	1.70 (1.40–2.07)	<0.001
Hypertension	1.64 (1.36–1.97)	<0.001	1.12 (0.92–1.37)	0.252
Diabetes	2.28 (1.79–2.91)	<0.001	1.45 (1.12–1.91)	0.005
Obesity	1.33 (1.12–1.57)	0.001	1.10 (0.88–1.38)	0.383
Smoking	1.38 (1.10–1.74)	0.006	1.35 (1.05–1.74)	0.020
NAFLD	1.45 (1.24–1.70)	<0.001	1.12 (0.92–1.35)	0.256
Triglyceride ⁺	1.30 (1.13–1.50)	<0.001	1.07 (0.89–1.28)	0.499
HDL cholesterol	0.88 (0.99–1.00)	0.031	1.00 (1.00–1.01)	0.374
Visceral fat area	1.01 (1.01–1.01)	<0.001	1.00 (0.89–1.28)	0.499
**MAFLD**				
Age	1.05 (1.04–1.06)	<0.001	1.05 (1.04–1.06)	<0.001
Sex	1.88 (1.61–2.21)	<0.001	1.84 (1.55–2.20)	<0.001
MAFLD	1.31 (1.12–1.53)	0.001	1.08 (0.91–1.28)	0.409
Smoking	1.31 (1.06–1.61)	0.011	1.36 (1.09–1.70)	0.007
Visceral fat area	1.01 (1.01–1.01)	<0.001	1.00 (1.00–1.01)	0.440

NAFLD, nonalcoholic fatty liver disease; OR, odds ratio; CI, confidence interval; MAFLD, metabolic dysfunction-associated fatty liver disease, ⁺ log transformed. NAFLD: adjusted for age, sex, body mass index, diabetes, hypertension, smoking, triglyceride l, HDL cholesterol, and visceral fat area. MAFLD: adjusted for age, sex, smoking, and visceral fat area.

**Table 3:** Multivariate analyses of the risk for colorectal adenoma according to sex.

	Women		Men	
NAFLD	OR (95% CI)		OR (95% CI)	
Hypertension	0.93 (0.63–1.39)	0.727	1.21 (0.96–1.52)	0.111
Diabetes	1.33 (0.76–2.31)	0.318	1.50 (1.11–2.03)	0.008
Obesity	0.93 (0.63–1.37)	0.711	1.13 (0.91–1.42)	0.270
Smoking	1.04 (0.41–2.64)	0.940	1.40 (1.07–1.83)	0.014
NAFLD	1.43 (1.01–2.03)	0.046	1.03 (0.82–1.28)	0.818
Visceral fat area	1.00 (1.00–1.01)	0.473	1.00 (0.99–1.00)	0.498
**MAFLD**				
MAFLD	1.55 (1.09–2.20)	0.015	0.97 (0.79–1.18)	0.722
Smoking	1.24 (0.52–2.99)	0.940	1.38 (1.09–1.74)	0.007
Visceral fat area	1.00 (0.99–1.01)	0.862	1.00 (1.00–1.01)	0.452

NAFLD, nonalcoholic fatty liver disease; OR, odds ratio; CI, confidence interval; MAFLD, metabolic dysfunction-associated fatty liver disease. NAFLD: adjusted for age, diabetes, hypertension, smoking, triglyceride, HDL cholesterol, and visceral fat area. MAFLD: adjusted for age, smoking, and visceral fat area.

**Table 4:** Multivariate analyses of the risk for colorectal adenoma by NAFLD/MAFLD with diabetes or obesity.

	Adenoma (%)	OR (95% CI)	*p* Value
No NAFLD	500/1901 (26.3%)	1 (Ref)	
NAFLD only	300/957 (31.3%)	1.07 (0.88–1.31)	0.489
NAFLD with diabetes	90/186 (48.4%)	1.75 (1.25–2.45)	0.001
No NAFLD	500/1901(26.3%)	1 (Ref)	
NAFLD only	198/575 (34.4%)	1.12 (0.90–1.40)	0.305
NAFLD with obesity	192/568 (33.8%)	1.12 (0.87–1.43)	0.386
No fatty liver disease	584/2162 (27.0%)	1 (Ref)	
Non-MAFLD steatosis	52/152 (34.2%)	1.29 (0.89–1.86)	0.176
MAFLD	374/1127 (33.2%)	1.09 (0.92–1.30)	0.319
No fatty liver disease	584/2162 (27.0)	1 (Ref)	
Non-MAFLD steatosis	52/152 (34.2)	1.29 (0.89–1.86)	0.174
MAFLD (lean)	15/52 (28.8)	1.03 (0.55–1.93)	0.918
MAFLD (overweight/obesity)	304/947 (32.1)	1.06 (0.88–1.28)	0.527
MAFLD (diabetes)	55/128 (43.0)	1.33 (0.91–1.95)	0.135

NAFLD, nonalcoholic fatty liver disease; OR, odds ratio; CI, confidence interval; MAFLD, metabolic dysfunction-associated fatty liver disease. NAFLD: adjusted for age, sex, smoking, triglyceride level, HDL cholesterol level, hypertension, visceral fat area, diabetes, and body mass index. MAFLD: adjusted for age, sex, smoking, and visceral fat area.

**Table 5:** The risk for colorectal adenoma in subjects with NAFLD/MAFLD according to advanced fibrosis.

	Adenoma (%)	OR (95% CI)	*p* Value
NAFLD with a low FIB-4 index	255/809 (31.5)	1 (Ref)	
NAFLD with a high FIB-4 index	132/327 (40.4)	1.38 (1.04–1.83)	0.027
MAFLD with a low FIB-4 index	304/947 (32.1)	1 (Ref)	
MAFLD with a high FIB-4 index	55/128 (43.0)	1.45 (1.13–1.96)	0.004

NAFLD, nonalcoholic fatty liver disease; OR, odds ratio; CI, confidence interval; FIB-4, Fibrosis-4 index. NAFLD: adjusted for sex, smoking, triglyceride level, HDL cholesterol level, diabetes, body mass index, hypertension, and visceral fat area. MAFLD: adjusted for sex, smoking, and visceral fat area.

## Data Availability

The data presented in this study are available on request from the corresponding author.

## Supplementary Materials

The following are available online at https://www.mdpi.com/article/10.3390/biomedicines9101401/s1, Table S1: Baseline characteristics according to NAFLD and MAFLD in men. Table S2: Baseline characteristics according to NAFLD and MAFLD in women.
