# Supplementary material for: The Risk of Colorectal Adenoma in Nonalcoholic or Metabolic-Associated Fatty Liver Disease
Source: Biomedicines. 2021 Oct 5;9(10):1401. doi: 10.3390/biomedicines9101401 (PMC8533199; doi:10.3390/biomedicines9101401)
Supplement: Supplementary file 1 [file biomedicines-09-01401-s001.zip › biomedicines-1393111-supplementary.pdf]

Supplementary Table S1. Baseline characteristics according to NAFLD and MAFLD in men.

	No NAFLD (N=946)	NAFLD (N=869)	*P*-value	No MAFLD (N=1233)	MAFLD (N=897)	*P*-value
Age (years)	52.8 ± 9.4	52.3 ± 8.6	0.256	52.3 ± 9.4	51.8 ± 8.5	0.224
Smoker, n (%)	167 (17.7)	168 (19.3)	0.357	252 (20.4)	191 (21.3)	0.631
Hypertension, n (%)	202 (21.4)	267 (30.7)	<0.001	268 (21.7)	297 (33.1)	<0.001
Diabetes, n (%)	82 (8.7)	146 (16.8)	<0.001	93 (7.5)	168 (18.7)	<0.001
BMI (kg/m^2)	23.4 ± 2.4	25.4 ± 2.4	<0.001	23.4 ± 2.4	25.7 ± 2.2	<0.001
BMI ≥25 (kg/m^2)	234 (24.7)	476 (54.8)	<0.001	297 (24.1)	535 (59.6)	<0.001
WC (cm)	85.2 ± 6.8	90.7 ± 6.5	<0.001	85.3 ± 6.8	91.6 ± 6.2	<0.001
SBP (mmHg)	117.4 ± 12.6	119.9 ± 12.5	<0.001	117.6 ± 12.6	120.8 ± 12.5	<0.001
DBP (mmHg)	75.9 ± 9.5	78.3 ± 9.4	<0.001	76.4 ± 9.5	79.0 ± 9.8	<0.001
AST (IU/L)	26.8 ± 19.0	29.6 ± 13.7	<0.001	27.1 ± 19.3	30.2 ± 13.6	<0.001
ALT (IU/L)	27.2 ± 22.1	37.2 ± 22.8	<0.001	27.8 ± 21.1	38.0 ± 23.0	<0.001
Total cholesterol (mg/dL)	192.1 ± 34.7	198.6 ± 39.5	<0.001	193.0 ± 34.30	198.4 ± 40.0	0.001
Triglyceride (mg/dL)	82 (58, 120)	120 (84, 165)	<0.001	83 (59, 120)	128 (88,174)	<0.001
HDL-cholesterol (mg/dL)	49.9 ± 10.1	45.7 ± 8.4	<0.001	50.1 ± 10.0	45.7 ± 8.5	<0.001
Fasting glucose (mg/dL)	95.5 ± 18.6	103.5 ± 23.4	<0.001	95.3 ± 17.4	104.7 ± 24.2	<0.001
HbA1c (mg/dL)	5.7 ± 0.6	5.9 ± 0.8	<0.001	5.6 ± 0.5	6.0 ± 0.9	<0.001

CRP (mg/dL)	0.2 ± 0.8	0.2 ± 0.9	0.507	0.2 ± 1.1	0.2 ± 0.7	0.483
Visceral fat area (cm^2)	80.8 ± 24.7	104.3 ± 22.5	<0.001	88.2 ± 24.4	104.7 ± 23.0	<0.001
Adenoma, n (%)	313 (33.1)	307 (35.3)	0.315	425 (34.5)	302 (33.7)	0.700

Data are shown as the mean ± SD. $^{+}$ median (interquartile range)

NAFLD, nonalcoholic fatty liver disease; MAFLD, metabolic dysfunction-associated fatty liver disease; BMI, body mass index; WC, waist circumference; SBP, systolic blood pressure; DBP, diastolic blood pressure; AST, aspartate aminotransferase; ALT, alanine aminotransferase; HDL, high-density lipid-cholesterol; HbA1C, glycated hemoglobin; CRP, c-reactive protein

Supplementary Table S2. Baseline characteristics according to NAFLD and MAFLD in women.

	No NAFLD (N=955)	NAFLD (N=274)	*P*-value	No MAFLD (N=1081)	MAFLD (N=230)	*P*-value
Age (years)	52.1 ± 9.2	55.9 ± 8.4	0.041	52.2 ± 9.2	56.6 ± 8.5	<0.001
Smoker, n (%)	26 (2.7)	6 (2.2)	0.625	27 (2.5)	5 (2.2)	0.773
Hypertension, n (%)	111 (11.6)	62 (22.6)	<0.001	129 (11.9)	57 (24.8)	<0.001
Diabetes, n (%)	28 (2.9)	40 (14.6)	<0.001	30 (2.8)	41 (17.8)	<0.001
BMI (kg/m^2)	21.6 ± 2.7	24.3 ± 3.4	<0.001	21.6 ± 3.2	25.1 ± 3.2	<0.001
BMI ≥25 (kg/m^2)	104 (10.9)	92 (33.6)	<0.001	114 (10.5)	97 (42.2)	<0.001
WC (cm)	78.5 ± 7.4	85.3 ± 8.2	<0.001	78.4 ± 7.2	87.3 ± 7.5	<0.001
SBP (mmHg)	112.1 ± 14.3	118.4 ± 14.1	<0.001	112.0 ± 14.2	120.7 ± 13.5	<0.001
DBP (mmHg)	69.7 ± 10.2	73.0 ± 9.5	<0.001	69.7 ± 10.2	74.4 ± 9.1	<0.001
AST (IU/L)	24.3 ± 8.7	27.9 ± 26.2	0.027	24.6 ± 9.9	28.9 ± 28.3	0.026
ALT (IU/L)	20.7 ± 11.1	29.2 ± 28.5	<0.001	21.3 ± 12.8	30.9 ± 30.6	<0.001
Total cholesterol (mg/dL)	196.7 ± 33.2	209.5 ± 37.9	<0.001	197.1 ± 33.4	210.4 ± 37.5	<0.001
Triglyceride (mg/dL)	59 (45, 82)	88 (63, 125)	<0.001	60 (45, 83)	98 (67,133)	<0.001
HDL-cholesterol (mg/dL)	57.5 ± 11.3	52.1 ± 10.1	<0.001	57.4 ± 11.2	50.9 ± 9.6	<0.001
Fasting glucose (mg/dL)	88.3 ± 13.7	98.8 ± 24.3	<0.001	88.3 ± 13.5	101.3 ± 25.8	<0.001
HbA1c (mg/dL)	5.6 ± 0.4	5.9 ± 0.8	<0.001	5.6 ± 0.4	6.0 ± 0.8	<0.001

CRP (mg/dL)	0.1 ± 0.5	0.3 ± 1.4	0.018	0.1 ± 0.5	0.4 ± 1.4	0.028
Visceral fat area (cm^2)	74.4 ± 23.3	94.7 ± 24.5	<0.001	74.4 ± 23.0	100.6 ± 21.6	<0.001
Adenoma, n (%)	187 (19.6)	83 (30.3)	<0.001	211 (19.5)	72 (31.3)	0.001

Data are shown as the mean ± SD. + median (interquartile range)

NAFLD, nonalcoholic fatty liver disease; MAFLD, metabolic dysfunction-associated fatty liver disease; BMI, body mass index; WC, waist circumference; SBP, systolic blood pressure; DBP, diastolic blood pressure; AST, aspartate aminotransferase; ALT, alanine aminotransferase; HDL, high-density lipid-cholesterol; HbA1C, glycated hemoglobin; CRP, c-reactive protein
